# Modeling of the Effect of Process Variations on a Micromachined Doubly-Clamped Beam

**DOI:** 10.3390/mi8030081

**Published:** 2017-03-05

**Authors:** Lili Gao, Zai-Fa Zhou, Qing-An Huang

**Affiliations:** Key Laboratory of MEMS of the Ministry of Education, Southeast University, Nanjing 210096, China; LilyGaoChina@gmail.com

**Keywords:** doubly-clamped beam, process variations, FE analysis, Bosch process, yield prediction

## Abstract

In the fabrication of micro-electro-mechanical systems (MEMS) devices, manufacturing process variations are usually involved. For these devices sensitive to process variations such as doubly-clamped beams, mismatches between designs and final products will exist. As a result, it underlies yield problems and will be determined by design parameter ranges and distribution functions. Topographical changes constitute process variations, such as inclination, over-etching, and undulating sidewalls in the Bosch process. In this paper, analytical models are first developed for MEMS doubly-clamped beams, concerning the mentioned geometrical variations. Then, finite-element (FE) analysis is performed to provide a guidance for model verifications. It is found that results predicted by the models agree with those of FE analysis. Assigning process variations, predictions for performance as well as yield can be made directly from the analytical models, by means of probabilistic analysis. In this paper, the footing effect is found to have a more profound effect on the resonant frequency of doubly-clamped beams during the Bosch process. As the confining process has a variation of 10.0%, the yield will have a reduction of 77.3% consequently. Under these circumstances, the prediction approaches can be utilized to guide the further MEMS device designs.

## 1. Introduction

Precise processing control has turned into an issue, owing to the mass production of micro-electro-mechanical systems (MEMS) devices and their increasingly complicated manufacturing processes. Discrepancies between initial designs and products deteriorate quickly with the feature size reductions. Even with the state-of-art fabrication techniques, process variations occur inevitably [1,2,3]. The process variations mainly include misalignment, footing as well as critical dimension (CD) loss [4], manifested as inclination, over-etching and undulating sidewalls in the Bosch process [5]. Typically, the effects of relative tolerances in MEMS devices are more severe than macro-scale products [1,6,7]. Relative manufacturing tolerances are alternatives to performance uncertainties. In addition, microstructures are commonly performed with nonlinear parallel plate electrostatic forces, which contributes to the complexity of problems. For such a reason, adequate methods are required due to the limitations in design rules and linear theories of MEMS [8].

Studies on process variations have achieved abundant results [9] in the scope of integrated circuits (ICs). However, these IC achievements cannot meet the whole needs of MEMS technologies. The conventionally used trial-and-error approach severely relies on the design-test cycle, which not only postpones the development cycle, but is also costly and time-consuming. Therefore, efforts have been made in the domain of MEMS devices analysis, for a better understanding of the impacts of process variations and also for reductions of performance variabilities at the design stage [10,11,12,13,14,15]. Islam et al. [10] conducted simulations and stress analysis on a fixed-fixed beam in electrostatic situations. Their results have reflected that changes in length and thickness tend to be more strictly controlled. Microbeam resonators are commonly utilized to detect or filter signals in MEMS. Due to manufacturing uncertainties, microbeam resonators undergo significant variability from initial designs. For example, Liu et al. [11] achieved tradeoff designs regarding multiple and conflicting design criteria, while Rong et al. [12] focused on multilayer structures while considering the first and second-order sensitivities of frequency. Mawardi et al. [13] utilized enumeration search and input–output relationships to get the governing parameters as well as a wide range for operating resonant frequency. In addition, magnetometers adopted multiphysics-based optimization and nonlinear situations [14], and gyroscopes focused on the packaging with double yield [15]. Except for unique device analysis, methods that are generally applicable have advanced the processing improvement further [3,16,17,18,19,20,21,22,23,24,25,26]. Mirzazaden et al. [16,17,18] investigated morphology uncertainties with reduced-order models through on-chip tests. Moreover, Shavezipur et al. [3,19], and Allen et al. [20] proposed the first-order second-moment (FOSM) and advanced FOSM reliability method, respectively, in a probabilistic way to obtain a linearized feasible region and maximize the yield. For those non-linear actuated MEMS devices, high fidelity optimization schemes have also been realized. To avoid the brute-force Monte Carlo (MC) scheme, Pfingsten et al. [21] considered a Bayesian Monte Carlo approach for yield estimation, with 90.0% computational savings but the same accuracy, compared with MC schemes. Vudathu et al. [22,26] applied a sensitivity analyzer for MEMS (SAM) by worst-case analysis, revealing the effects of parametric variations on performance and yield. Achievements have been reached to get a balance between precision and calculation—for example, the Sigma-Point approach applied to MEMS resonators with four orders of magnitude faster than MC [23], the generalized polynomial chaos (GPC) framework to handle stochastic coupled electromechanical analysis with the same precision and one order of magnitude faster compared with MC [24], and the Taguchi parameter design and statistical process-control method to minimize variability in performance response to fluctuations [25].

However, little work has been carried out for detailed analysis of specific processing steps. This paper intends to explore the effect of process variations on the resonance frequency of doubly-clamped beams, under the Bosch target processing environment. The commercial code ANSYS (11.0) [27] guides the verifications of the presented methods. The results suggest that with assigned process variations, structure performance and yield can be predicted. On the other hand, given design specifications, reasonable suggestions can be made for parameter error ranges under process variations.

## 2. Process Variations

**Figure 1:**
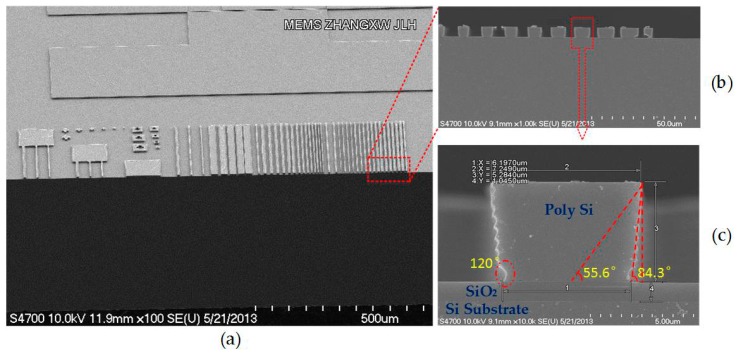
Scanning electron microscope (SEM) cross section for deep reactive ion etching (DRIE) beams: (**a**) top view of beam array; (**b**) side-view of the beams labeled in (**a**); (**c**) side-view of the single beam labeled in (**b**), where the footing effect reflects as an arc angle approximating 120°, the inclination angle of 84.3° and 55.6° in the worst-case.

Marked as a highly anisotropic etching process with high aspect ratios, deep reactive-ion etching (DRIE) is employed to create deep penetration, steep-sided holes and trenches in wafers or substrates. The Bosch process is one of the high-rate DRIE technologies, capable of fabricating 90° vertical walls theoretically [28,29,30]. The Bosch process alternates between isotropic plasma etching and deposition of a passivation layer, also called pulsed or time-multiplexed etching. The etching–deposition procedures will be repeated until all of the demands are satisfied. However, it is hard to obtain a sidewall precisely vertical to the substrate. Morphology features like inclinations, undulating ripples as well as over-etching are inevitable and critical, which are called the trapezium effect, the ripple effect and footing effect in the following, respectively. The variations induced by these effects manifest roughly as planar sizes (dominated by photolithography and etching processes), planar position offset (dominated by alignment) and vertical sizes (dominated by thickness variations of thin films or the substrate). An ideal beam is a cuboid structure with a length dominated as l, a width as w, and the thickness as h. The cross section was supposed to be a standard rectangle, while, in fact, it appeared as the side-view given in Figure 1a–c. The beams illustrated in Figure 1 are fabricated by DRIE technology, with the sidewall inclination around 84.3°, the undulating ripples about 120°, and the over-etching circled in red of Figure 1c.

**Figure 2:**
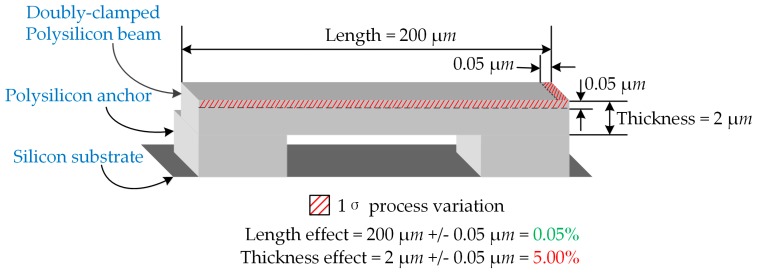
Side-view of a doubly-clamped beam section, assumed with length 200±0.05 μm and thickness 2±0.05 μm. The red part stands for manufacturing process variations, marked as 0.05% and 5.0% on beam length and thickness, respectively.

To reveal the significance of manufacturing process variations, a simplified doubly-clamped beam is illustrated in Figure 2. The thickness and length of the beam are assumed to undergo the same manufacturing process variation as 0.05 μm. Thus, for a 200 μm long and 2 μm thick beam, the relative error for the length equals 0.05%, while it is 5.0% for the thickness case. The parameter thickness is obviously more sensitive to process variations. Combined with the usually quadruple relationship of length in a beam’s frequency, the relative error diminishes to 0.0006%. This finding reveals that the length variation can be ignored in certain cases to simplify the analysis models.

## 3. Problem Solution

Studies on MEMS devices have pointed out that manufacturing process variations have a close relationship with performance drift and device failure [22,25,31]. As the basic element in MEMS, the resonant frequency of the doubly-clamped beam underlies the majority of engineering designs. Assuming the section as a plane, the doubly-clamped beam can be treated as an Euler–Bernoulli beam. Without initial buckling, the differential equation for lateral oscillation can be expressed as

(1) $$\bar{EI}\frac{\partial^{4}z\left(x,t\right)}{\partial x^{4}}-\bar{\sigma A}\frac{\partial^{2}z\left(x,t\right)}{\partial x^{2}}=-\bar{\rho A}\frac{\partial^{2}z\left(x,t\right)}{\partial t^{2}},$$

where $\bar{EI}$ is the bending stiffness, $\bar{\rho A}$ is the linear density, $\bar{\sigma A}$ is the axial load, and z(x,t) is the displacement along the *z*-axis. Ignoring the residual stress, the resonant frequency of the doubly-clamped beam approximates as [32,33]

(2) $$f_{i}=\frac{1}{2\pi }{\left(k_{i}l\right)}^{2}\sqrt{\frac{\bar{EI}}{\bar{\rho A}l^{4}}},$$

in which $k_{i}l$ stands for the coefficient of the *i*th mode of vibration, and the first three values as $k_{1}l=4.730$, $k_{2}l=7.853$, $k_{3}l=10.996$.

### 3.1. Effect of a Single Factor

Apart from geometrical size errors that can be presented in the behavior equations (refer to Appendix A and Appendix B), morphology changes play an important role in the variability of devices’ performance and yield. Appropriate models are needed to reflect the main causes that result from manufacturing uncertainties. As stated in Section 2, process variations are mainly rooted in the trapezium, footing and ripple effect. These effects primarily occur in the Bosch process, Reactive ion etching (RIE) for silicon-on-insulator (SOI) structures, as well as time-multiple-deep deposition (TMDE), respectively. More details on the model building are shown in Appendix A.

**Figure 3:**
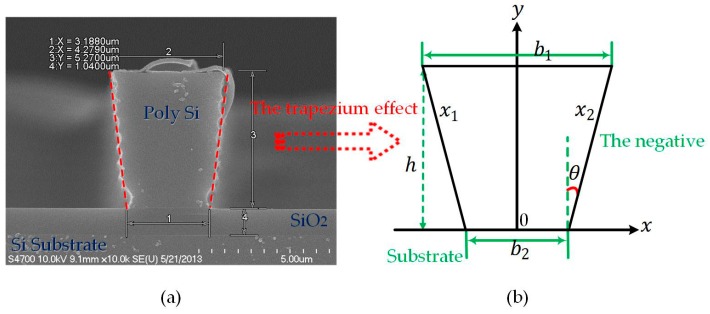
The trapezium effect of a doubly-clamped beam: (**a**) SEM side-view, fabricated by the Bosch process and un-released; (**b**) the coordinate sketch-map for the negative.

The cross section of the beam has been transformed from an ideal rectangle to a trapezoid profile due to process variations. This phenomenon is defined as the trapezium effect, which can be divided into the positive trapezium effect (the trapezoid angle θ>0) and the negative trapezium effect (the trapezoid angle θ<0). Figure 3 represents the latter, where Figure 3a denotes the section of a doubly-clamped beam. Figure 3b extracts its cross section models and parameters in the coordinate system. In such a case, the resonant frequency changes into:

(3) $$f_{t}=\frac{{\left(k_{i}l\right)}^{2}}{6\pi }\sqrt{\frac{Eh^{2}\left(b_{1}^{2}+b_{2}^{2}+4b_{1}b_{2}\right)}{2\rho l^{4}{\left(b_{1}+b_{2}\right)}^{2}}}.$$

**Figure 4:**
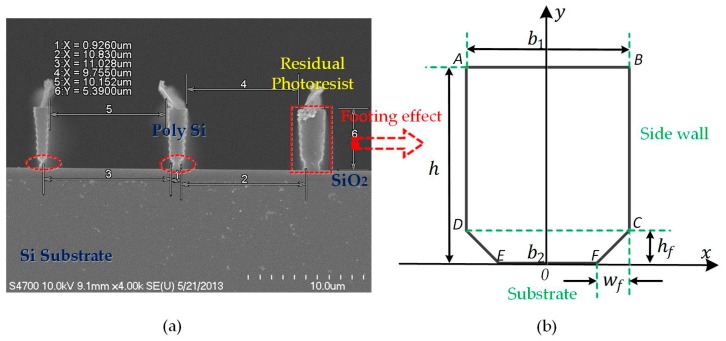
Footing effect of a doubly-clamped beam: (**a**) SEM side-view, fabricated by the Bosch process and un-released; (**b**) the coordinate sketch-map for the structure circled in red in (**a**).

The footing effect usually shows up in the RIE/DRIE process of SOI structures. It causes inhomogeneous distributions of the mass and stiffness, even making the structure part collapse. The over-etching height $h_{f}$ and horizontal over-etching width $w_{f}$ are viewed as the key elements, as demonstrated in Figure 4. Without careful processing control or wide enough width, the structure is prone to crash down, inferred from Figure 4a. The footing effect changes the resonant frequency into:

(4) $$f_{f}=\frac{{\left(k_{i}l\right)}^{2}}{2\pi }\sqrt{\frac{E\left(\frac{1}{3}b_{1}h^{3}-\frac{1}{6}w_{f}h_{f}^{3}-\frac{{\left(\frac{1}{2}b_{1}h^{2}-\frac{1}{3}w_{f}h_{f}^{2}\right)}^{2}}{b_{1}h-w_{f}h_{f}}\right)}{\rho l^{4}\left(b_{1}h-w_{f}h_{f}\right)}}.$$

**Figure 5:**
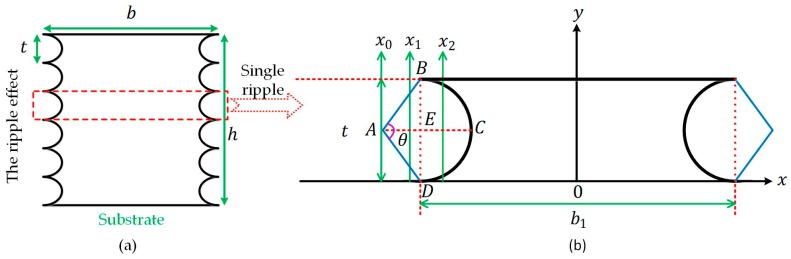
The ripple effect of a doubly-clamped beam: (**a**) a schematic diagram of the model; (**b**) the coordinate sketch-map of single ripple as one unit.

Ripples are presented on the rough side walls of structures with high aspect ratios, owing to the TMDE technology. It is defined as the ripple effect, referring to the simplified model in Figure 5a,b. Given the ripple arc ranging from 30° to 180° and single ripple height from 0.1 μm to 1 μm, simulations in ANSYS have suggested that the deciding element in the ripple effect is single ripple height, rather than ripple arc with an error within 2.0%. Therefore, single ripple height, t, can be treated as a key when dealing with the ripple effect.

In such a case, the resonant frequency changes into:

(5) $$f_{r}=\frac{{\left(k_{i}l\right)}^{2}}{2\pi }\sqrt{\frac{E\left(-\frac{3\sqrt{3}+8\pi }{144}ht^{3}-\frac{2\pi -3\sqrt{3}}{72}h^{3}t+\frac{1}{12}b_{1}h^{3}\right)}{\rho l^{4}\left(b_{1}h-\frac{2\pi -3\sqrt{3}}{6}ht\right)}},$$

where the meaning of the symbols is marked in Figure 5.

### 3.2. Effect of Multiple Factors

**Figure 6:**
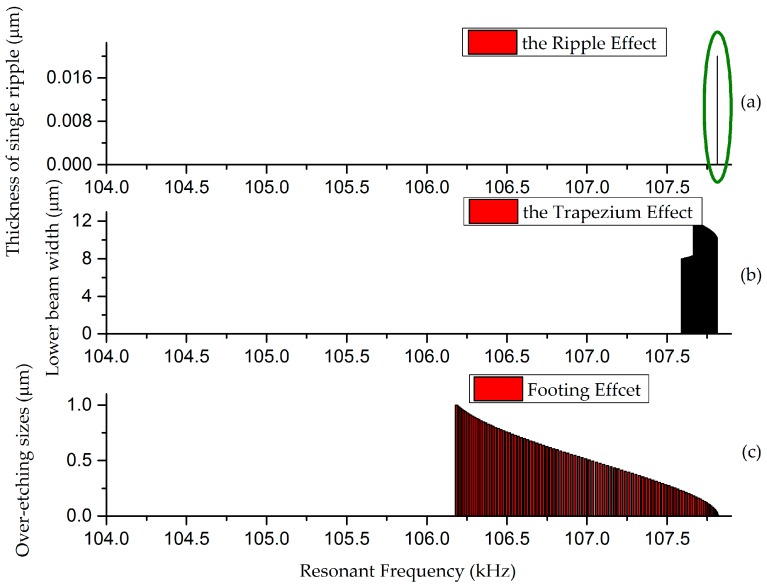
Sensitivity analysis on the effects influencing the resonant frequency of doubly-clamped beams. (**a**) is the result with considering the ripple effect. The narrow band circled by green equals the working part. (**b**,**c**) are cases for the trapezium effect and footing effect, where the cross section is treated as isosceles trapezoid in (**b**) and the over-etching sizes longitudinally and laterally are equal in (**c**).

Sensitivity analyses on the effects mentioned above are conducted, shown in Figure 6. With accurate processing control and mature techniques, single arc height can be restricted to be less than 0.01 μm so that the ripple effect can be diminished, as shown in Figure 6a. Under these circumstances, models can be simplified into two critical effects: the trapezium effect and footing effect. Thus, when side walls are thought to be smooth, the resonant frequency of doubly-clamped beams can be expressed as:

(6) $$f_{tf}=\frac{{\left(k_{i}l\right)}^{2}}{2\pi }\sqrt{\frac{E\left(\frac{h^{3}}{36}\cdot \frac{b_{1}^{2}+b_{2}^{2}+4b_{1}b_{2}}{b_{1}+b_{2}}-2\left(\frac{w_{f}h_{f}^{3}}{36}+\frac{w_{f}h_{f}}{2}{\left(\frac{h_{f}}{3}-\frac{h\left(2b_{1}+b_{2}\right)}{3\left(b_{1}+b_{2}\right)}\right)}^{2}\right)\right)}{\rho l^{4}\left(\frac{b_{1}+b_{2}}{2}h-w_{f}h_{f}\right)}}.$$

**Figure 7:**
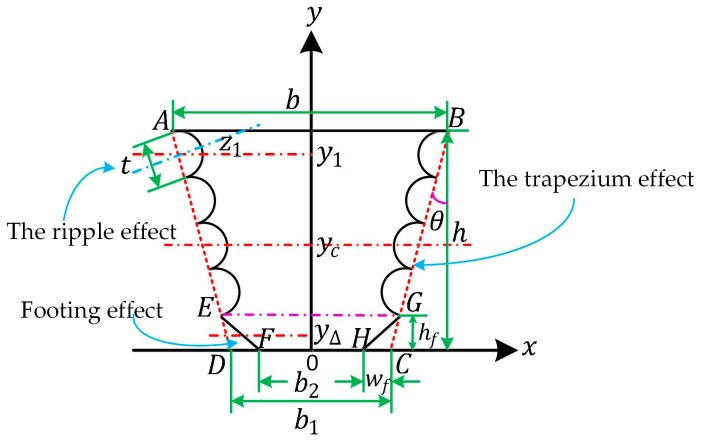
The coordinate sketch-map for the cross section of the doubly-clamped beam considering the three effects simultaneously (only four ripples for illustration).

However, side walls cannot be treated as smooth all the time. Models containing the three effects simultaneously are essential. The corresponding coordinate is illustrated as Figure 7, where $b_{1}=b+2h\text{tan}\theta$, $b_{2}=b_{1}-2w_{f}$. When the gap between beams is wide enough, like 6 μm or wider, the cross section can be assumed to be continuous, repeated and symmetrical. Assumptions can be raised that the central axis of the cross section equals the central axis of the trapezoid *ABCD*, which means $y=y_{c}$, and that the ripple is a semicircle. The moment of inertia, relative to central axis $y=y_{1}$, equals the condition of axis $z_{1}$, approximately. Axis $z_{1}$ is in the direction of the semicircle radius and perpendicular to the side waist of trapezoid ABCD, when the semicircle vibrates longitudinally.

The above results suggest that the resonant frequency changes into (refer to Appendix B for more details on model building):

(7) $$f_{tfr}=\frac{{\left(k_{i}l\right)}^{2}}{2\pi }\sqrt{\frac{E\left(\frac{h^{3}}{36}\cdot \frac{b^{2}+b_{1}^{2}+4bb_{1}}{b+b_{1}}-2\left(\frac{w_{f}h_{f}^{3}}{36}+\frac{A_{f}}{2}{\left(\frac{h_{f}}{3}-\frac{2b+b_{1}}{3\left(b_{1}+b\right)}h\right)}^{2}\right)-2\overset{N}{\underset{i=1}{\sum }}\left(\frac{1}{2}\cdot \frac{1}{64}\pi r^{4}+\frac{\pi r^{2}}{2}{\left(y_{1}-i*2r\text{cos}\theta -y_{c}\right)}^{2}\right)\right)}{\rho l^{4}\left(\frac{\left(b_{1}+b\right)h}{2}-w_{f}h_{f}-N\pi r^{2}\right)}}$$

## 4. Analysis and Results

Geometric features of MEMS devices usually do not comply with the design value, with the typical error around 5.0% [34,35] during the manufacturing processes. Design parameters for the doubly-clamped beam are listed in Table 1.

Multi-field models are always complicated for the complex mechanism of MEMS devices. The situation deteriorates with stochastic manufacturing uncertainties. Leaving the cost alone, with repeated adjustment or tape-out of test structures, only a small percent of the data is acceptable. Due to the lack of manufacturing data, the trial-and-error method is not optimal. Adequate models underlying process variations should be developed.

**Figure 8:**
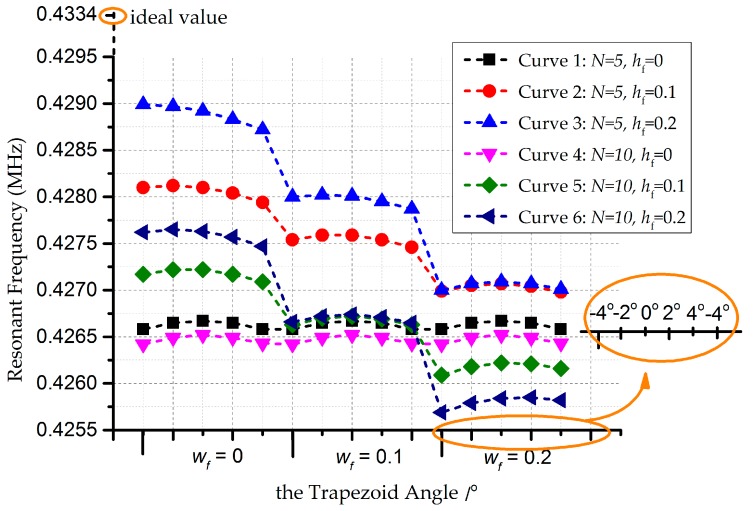
Change curves of the resonance frequency for the modified model of the doubly-clamped beam. The *x* coordinate is divided into three intervals, each of which is denoted as magnified in the orange circle.

Simulations are conducted in ANSYS WORKBENCH 14.5 (ANSYS, Pittsburgh, PA, USA), with the solver SOLID45 (ANSYS, Pittsburgh, PA, USA) [27]. The mesh method uses tetrahedrons with patch conforming. Lateral vibrations are considered to perform model verifications, as shown in Figure 8. The number of ripples is supposed to be 5 and 10, in order to simplify the validations. Assuming small variation ranges in key elements of the trapezium and footing effect, analyses of curves in Figure 8 are carried out. The resonant frequency is found to be in direct proportion to the number of ripples *N*, with improvement in stable behavior along with larger *N*. Morever, the frequency manifests a reverse proportion to the angle, undergoing serious shifts in the wake of deteriorative footing effect.

**Figure 9:**
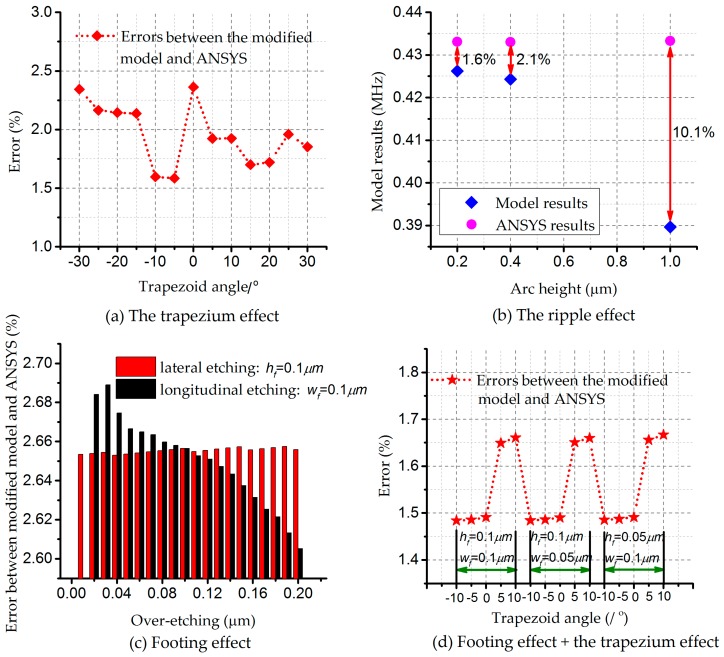
Comparisons between modified models and ANSYS while considering models related to Equations (3)–(6) (the errors along the vertical axis defined as: Error = |model results—ANSYS results|/model results): (**a**) the trapezium effect; (**b**) the ripple effect; (**c**) the footing effect, the red for lateral over-etching and black for over-etching longitudinally; and (**d**) the footing effect and the trapezium effect.

In light of the above development, comparisons between modified models and ANSYS are conducted, with consideration of single effects, respectively. Figure 9a describes errors under the trapezium effect, with the bias less than 2.5%. It turns out that the model and simulations share a similar trend. Figure 9b states the situation for the ripple effect. As the situation for a high value of the arc height rarely occurs during processing, the result with an error of 10.1% is not accurate or applicable for further study. The first two results are receivable in general, limiting the errors within 2.0%. Confining errors within 3%, over-etching longitudinally introduces more variabilities in resonant frequency, referred to in Figure 9c. Complicated structures will result in larger differences. Analyses are conducted in Figure 9d to explain the footing and trapezium effects. In pursuit of less variabilities in frequency, the negative trapezium effect is proved to be effective. In addition, the footing effect occupies the dominant position, compared with the trapezium effect, according to Figure 6b,c.

**Figure 10:**
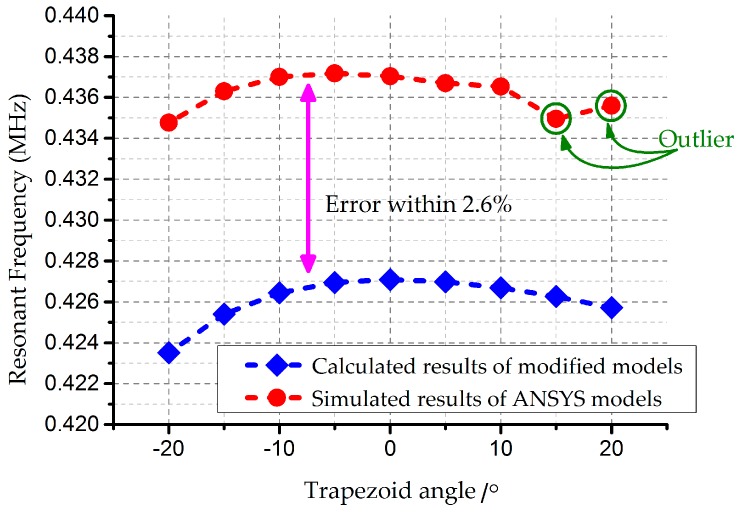
Change curves of the resonance frequency for the doubly-clamped beam. Process variations are set as ±0.5 μm. The dots in green circles are outliers of the simulated results.

Furthermore, comparisons have been established between modified models and FE analysis. The number of ripples *N* is assigned to 10, while $w_{f}=h_{f}=0.2\;\mu m$ in the footing effect. The curves share an error within 2.6%, according to Figure 10. The outliers in the upper right corner of Figure 10 suggest biases of ANSYS simulations. This occurrence is attributed to the unpractical assumptions that angles in the positive trapezium effect can be 20°, in which case the principles of Timoshenke beams cannot be applied directly. However, the assumptions give credit to the negative case for the existence of the footing effect. The two curves trend similarly in general, which confirms the acceptability of the modified models.

Direct Monte Carlo (MC) simulations are performed based on the modified models. Yield is defined as a factor of the proportion falling into the same distribution range. Doubly-clamped beams with 400 μm length, 10 μm width and 4 μm thickness are raised as an example in these simulations. Hypotheses are proposed that all the parameters concerned comply with the Gaussian’s distribution, along with the same process variation ±0.5 μm. The sampling numbers for MC simulations range from 100,000 to 1,000,000. The relative error for frequency turns out to be around 7.9% and an angle of around ±7° when considering the trapezium effect. The resonant frequency reduces from 22.7% to 33.0% while the yield decreases to nearly 67.0% under the footing effect. When doubling the numbers of ripples, relative errors for the resonant frequency can be improved about 1.0%.

## 5. Conclusions

This paper has considered the problems existing in the Bosch process and their negative influences on MEMS doubly-clamped beam performance. Modified models of doubly-clamped beams were built, with consideration for the trapezium, footing, and ripple effects respectively and simultaneously. The relative performance error was restricted to 10.0%, with a yield of about 77.3% if process variations were assumed to be in the same range. FE verifications have been performed to validate the models built in this study, indicating that the heavy simulation work can be substituted in some cases by applying the models.

The model results can be viewed as the guidance for design cycle optimizations. Designers can directly figure out the key elements in the etching process by reconsidering the design sizes and shapes, and eventually compensate the errors brought by process variations to improve the yield.

Other critical elements such as residual stress, gaps between beams, or variations in Young’s modulus were not considered in this paper and will be discussed in future work. Moreover, diversified distribution forms such as quasi-Gaussian can be applied in MC methods and will be the focus of future research.

## Figures and Tables

**Table 1:** Design parameters for a doubly-clamped beam.

Structure Parameters	Values
Beam length l/μm	200
Beam width b/μm	4
Beam thickness h/μm	2
Young’s modulus E/GPa	158
Material density $\rho /\mathrm{kg}/\mu m^{3}$	$2.23\times 10^{-15}$

**Figure B1:**
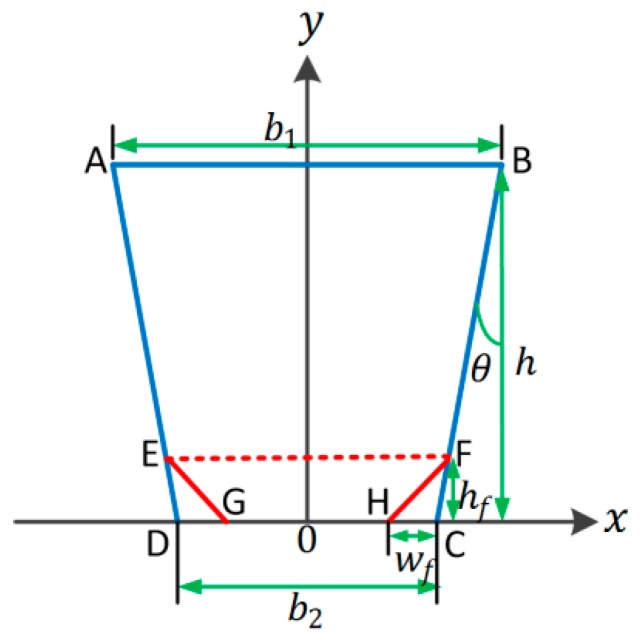
The coordinate sketch-map for the cross section of a doubly-clamped beam while considering the footing effect and the trapezium effect.

**Figure B2:**
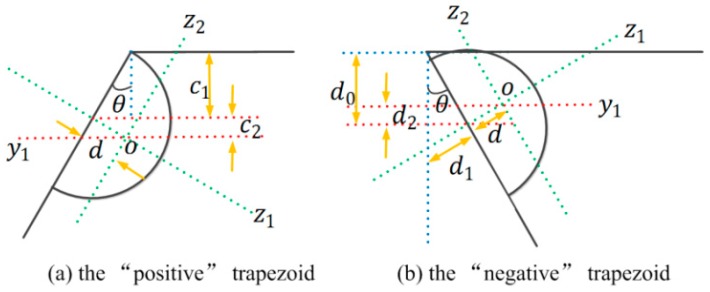
Coordinate sketch-map for the first arc. (**a**) the positive trapezoid (the upper length lower than the lower length) while the trapezoid angle θ>0; (**b**) the negative one and θ<0.

## Appendix A. Single Factor Effect

### Appendix A.1. The Trapezium Effect

During the DRIE processes, the ideal rectangle profile of a beam can be transferred to a trapezoid profile due to process variations. Usually, the cases are divided into the positive trapezium effect (the trapezoid angle θ>0) and the negative trapezium effect (the trapezoid angle θ<0), shown in Figure 3. For the ideal doubly-clamped beam, the resonance frequency can be expressed as:

(A1) $$f=\frac{{\left(k_{i}l\right)}^{2}}{4\pi }\sqrt{\frac{Ew^{2}}{3\rho l^{4}}}.$$

Inducing *x*-*y* coordinates (the same way for the positive), the upper width $b_{1}$ is:

(A2) $$b_{1}=b_{2}-2h\cdot \tan\theta .$$

The neutral axis could be denoted by $y_{na}$, along with the linear equations of $x_{1}$ and $x_{2}$, that is:

(A3) $$y_{na}=\frac{S_{z}}{A_{t}}=\frac{2b_{1}+b_{2}}{3\left(b_{1}+b_{2}\right)}h.$$

Here, $S_{z}$ is the static moment, and $A_{t}$ is the current cross-sectional area.

Then, the moment of inertia is expressed as:

(A4) $$I_{t}=\int_{A}y^{2}dA==\frac{h^{3}\left(b_{1}^{2}+b_{2}^{2}+4b_{1}b_{2}\right)}{36\left(b_{1}+b_{2}\right)}.$$

Thus, the resonant frequency of the doubly-clamped beam for the modified model considering the trapezoid effect changes into:

(A5) $$f_{t}=\frac{{\left(k_{i}l\right)}^{2}}{6\pi }\sqrt{\frac{Eh^{2}\left(b_{1}^{2}+b_{2}^{2}+4b_{1}b_{2}\right)}{2\rho l^{4}{\left(b_{1}+b_{2}\right)}^{2}}.}$$

### Appendix A.2. The Footing Effect

The critical element in the footing effect is the over-etching height $h_{f}$ and horizontal over-etching width $w_{f}$, as shown in Figure 4. According to the properties for the moment of inertia, the moment of inertia relative to the neutral axis for the cross section *ABCFED* can be expressed as:

(A6) $$I_{ABCFED}^{\prime }=I_{ABCD}^{\prime }+I_{CDEF}^{\prime },$$

where

(A7) $$I_{ABCD}^{\prime }=\frac{1}{12}b_{1}{\left(h-h_{f}\right)}^{3},$$

(A8) $$I_{CDEF}^{\prime }=\frac{h_{f}^{3}}{36\left(b_{1}-w_{f}\right)}\left(3b_{1}^{2}-6b_{1}w_{f}+2w_{f}^{2}\right).$$

Then, regards to the parallel-axis theorem, the moment of inertia relative to the neutral axis $y_{c}$ for the cross section *ABCFED* becomes

(A9) $$I_{f}=I_{ABCFED}=I_{ABCD}+I_{CDEF}=I_{ABCD}^{\prime }+b_{1}\left(h-h_{f}\right){\left(y_{c}-y_{1}\right)}^{2}+I_{CDEF}^{\prime }+h_{f}\left(b_{1}-w_{f}\right){\left(y_{c}-y_{2}\right)}^{2}.$$

Here, $y_{1}$ and $y_{2}$ denote the neutral axis of *ABCD* and *CDEF*, relative to each own centroid, respectively, and

(A10) $$y_{1}=\frac{h+h_{f}}{2},\;y_{2}=\frac{3b_{1}-2w_{f}}{26b_{1}-6w_{f}}h_{f},\;y_{c}=\frac{\frac{1}{2}b_{1}h^{2}-\frac{1}{3}w_{f}h_{f}^{2}}{b_{1}h-w_{f}h_{f}}.$$

Thus, the frequency of the doubly-clamped beam for the modified model considering the footing effect changes into:

(A11) $$f_{f}=\frac{{\left(k_{i}l\right)}^{2}}{2\pi }\sqrt{\frac{E\left(\frac{1}{3}b_{1}h^{3}-\frac{1}{6}w_{f}h_{f}^{3}-\frac{{\left(\frac{1}{2}b_{1}h^{2}-\frac{1}{3}w_{f}h_{f}^{2}\right)}^{2}}{b_{1}h-w_{f}h_{f}}\right)}{\rho l^{4}\left(b_{1}h-w_{f}h_{f}\right)}}.$$

### Appendix A.3. The Ripple Effect

The key element of the ripple effect is the height of the ripple, almost nothing to do with the arc of the ripple. Therefore, the height of the ripple, t, is taken as an impact factor of the ripple effect shown as Figure 5. The impacts on frequency mainly arise from t, and the central angle is chosen as $\theta =60^{{}^{\circ}}$, according to the statistical results of tapeout (for the further model optimization, θ could be treated as an independent variable). Similar to the knowledge of moment of inetia, the cross section regarding x=0 under the ripple effect is the sum of each unit, depicted in Figure 5. Symetrically, the moment of inertia of each unit stays the same, regarding the axis of x=0, denoted as $I_{0}$. Here, we have AB=AD=BD=r=t, $AE=\frac{\sqrt{3}}{2}r$, $CE=r-\frac{\sqrt{3}}{2}r$.

With a series of calculations, the moment of inertia relative to the neutral axis itself for one unit is:

(A12) $$I_{0}=\frac{b_{1}t^{3}}{12}+\frac{\sqrt{3}-4\pi }{48}t^{4}.$$

Similarly, the sum of the moment of inertia under the ripple effect is expressed as:

(A13) $$I_{r}=\sum I_{i}=\sum_{i=1}^{i=N}\left(I_{0}+{\left(\frac{h}{2}-\frac{2i-1}{2}t\right)}^{2}\left(b_{1}t-\frac{2\pi -3\sqrt{3}}{6}t^{2}\right)\right)=-\frac{3\sqrt{3}+8\pi }{144}ht^{3}-\frac{2\pi -3\sqrt{3}}{72}h^{3}t+\frac{1}{12}b_{1}h^{3}.$$

Finally, the frequency of the doubly-clamped beam for the modified model considering the ripple effect changes into:

(A14) $$f_{r}=\frac{{\left(k_{i}l\right)}^{2}}{2\pi }\sqrt{\frac{E\left(-\frac{3\sqrt{3}+8\pi }{144}ht^{3}-\frac{2\pi -3\sqrt{3}}{72}h^{3}t+\frac{1}{12}b_{1}h^{3}\right)}{\rho l^{4}\left(b_{1}h-\frac{2\pi -3\sqrt{3}}{6}ht\right)}}.$$

## Appendix B. Multiple Factors Effect

### Appendix B.1. The Ripple Effect and Footing Effect

As the moment of inertia is essential for model building (the same sequence as it in Appendix A1), the moment of inertia can de deduced from the trapezium effect. If the over-etching part arising from the footing effect is neglected compared with the part in the trapezium effect, the transversal change of the neutral axis due to the footing effect could also be ignored. Applying the corresponding coordinate in Figure 6 and Figure B1, the moment of inertia for the cross section is written as:

(B1) $$I_{tf}=I_{ABCD}-I_{DEG}-I_{CFH}=\frac{h^{3}}{36}\cdot \frac{b_{1}^{2}+b_{2}^{2}+4b_{1}b_{2}}{b_{1}+b_{2}}-2\left(\frac{w_{f}h_{f}^{3}}{36}+\frac{w_{f}h_{f}}{2}{\left(\frac{h_{f}}{3}-\frac{h\left(2b_{1}+b_{2}\right)}{3\left(b_{1}+b_{2}\right)}\right)}^{2}\right),$$

where $s_{tf}=\frac{b_{1}+b_{2}}{2}h-w_{f}h_{f}$. The frequency of the doubly-clamped beam for the modified model considering the footing effect and the trapezium effect changes into:

(B2) $$f_{tf}=\frac{{\left(k_{i}l\right)}^{2}}{2\pi }\sqrt{\frac{E\left(\frac{h^{3}}{36}\cdot \frac{b_{1}^{2}+b_{2}^{2}+4b_{1}b_{2}}{b_{1}+b_{2}}-2\left(\frac{w_{f}h_{f}^{3}}{36}+\frac{w_{f}h_{f}}{2}{\left(\frac{h_{f}}{3}-\frac{h\left(2b_{1}+b_{2}\right)}{3\left(b_{1}+b_{2}\right)}\right)}^{2}\right)\right)}{\rho l^{4}\left(\frac{b_{1}+b_{2}}{2}h-w_{f}h_{f}\right)}.}$$

### Appendix B.2. The Ripple Effect, the Footing Effect, and the Ripple Effect

The corresponding coordinate for the three effects is shown in Figure 6, where $b_{1}=b+2h\text{tan}\theta$, and $b_{2}=b_{1}-2w_{f}$. With the assumptions proposed above, the area and moment of inertia for the cross section are written as:

(B3) $$A=\frac{\left(b_{1}+b\right)h}{2}-w_{f}h_{f}-N\pi r^{2},$$

(B4) $$I=I_{ABCD}-I_{DEF}-I_{CGH}-\sum I_{arc}.$$

In addition, it is easy to get:

(B5) $$I_{ABCD}=\frac{h^{3}}{36}\cdot \frac{b^{2}+b_{1}^{2}+4bb_{1}}{b+b_{1}},$$

(B6) $$I_{f}=I_{DEF}+I_{CGH}=2\left(\frac{w_{f}h_{f}^{3}}{36}+\frac{A_{f}}{2}{\left(\frac{h_{f}}{3}-\frac{2b+b_{1}}{3\left(b_{1}+b\right)}h\right)}^{2}\right).$$

Models should be developed to analyze arc coordinates for the solution of $I_{arc}$ when the trapezoid angle θ>0 or θ<0. The key to the arc coordinates is the position of the first arc (see Figure B2).

In Figure B2a, $z_{1}$ and $z_{2}$ are the neutral axes of the semicircle itself, by the point of their intersection o, and $y_{1}$ is parallel to $y_{c}$. Consequently, the coordinate of $y_{1}$ is $y_{1}=h-\frac{r}{\text{cos}\theta }-d\text{tan}\theta$. For the condition in Figure B2b, $y_{1}=h-\frac{r}{\text{cos}\theta }+\left(d+r\text{tan}\theta \right)\text{sin}\theta$. With the assumptions above, the moment of inertia for the semicircle relative to neutral axis $z_{1}$ equals $I_{0}=\frac{1}{2}\cdot \frac{1}{64}\pi r^{4}$, and then the moment of inertia relative to the cross section for the *i*th arc becomes

(B7) $$I_{i}=\frac{1}{2}\cdot \frac{1}{64}\pi r^{4}+\frac{\pi r^{2}}{2}{\left(y_{1}-i*2r\text{cos}\theta -y_{c}\right)}^{2}.$$

Therefore,

(B8) $$I=I_{ABCD}-I_{f}-2\sum_{i=1}^{n}I_{i}.$$

Then, the frequency of the doubly-clamped beam for the modified model considering three main effects changes into

(B9) $$f_{tfr}=\frac{{\left(k_{i}l\right)}^{2}}{2\pi }\sqrt{\frac{E\left(\frac{h^{3}}{36}\cdot \frac{b^{2}+b_{1}^{2}+4bb_{1}}{b+b_{1}}-2\left(\frac{w_{f}h_{f}^{3}}{36}+\frac{A_{f}}{2}{\left(\frac{h_{f}}{3}-\frac{2b+b_{1}}{3\left(b_{1}+b\right)}h\right)}^{2}\right)-2\sum_{i=1}^{N}\left(\frac{1}{2}\cdot \frac{1}{64}\pi r^{4}+\frac{\pi r^{2}}{2}{\left(y_{1}-i*2r\text{cos}\theta -y_{c}\right)}^{2}\right)\right)}{\rho l^{4}\left(\frac{\left(b_{1}+b\right)h}{2}-w_{f}h_{f}-N\pi r^{2}\right)}.}$$
